# Gastrointestinal Hormones in Healthy Adults: Reliability of Repeated Assessments and Interrelations with Eating Habits and Physical Activity

**DOI:** 10.3390/nu13113809

**Published:** 2021-10-26

**Authors:** Silke M. Wortha, Katharina A. Wüsten, Veronica A. Witte, Nicole Bössel, Wolfram Keßler, Antje Vogelgesang, Agnes Flöel

**Affiliations:** 1 Department of Neurology, University Medicine Greifswald, 17475 Greifswald, Germany; silkemaria.wortha@med.uni-greifswald.de (S.M.W.); Annick.Wuesten@t-online.de (K.A.W.); nb152022@uni-greifswald.de (N.B.); antje.vogelgesang@uni-greifswald.de (A.V.); 2 Department of Cognitive Neurology, University of Leipzig Medical Center, 04103 Leipzig, Germany; witte@cbs.mpg.de; 3 Max Planck Institute for Cognitive and Brain Sciences, 04103 Leipzig, Germany; 4 Department of General, Visceral, Thoracic and Vascular Surgery, University Medicine, 17475 Greifswald, Germany; wolfram.kessler@med.uni-greifswald.de; 5 German Centre for Neurodegenerative Diseases (DZNE), Site Rostock/Greifswald, 17489 Greifswald, Germany

**Keywords:** intra-class correlation, reliability, GI hormones, healthy participants

## Abstract

*Background:* Gastrointestinal hormones (GIHs) are crucial for the regulation of a variety of physiological functions and have been linked to hunger, satiety, and appetite control. Thus, they might constitute meaningful biomarkers in longitudinal and interventional studies on eating behavior and body weight control. However, little is known about the physiological levels of GIHs, their intra-individual stability over time, and their interaction with other metabolic and lifestyle-related parameters. Therefore, the aim of this pilot study is to investigate the intra-individual stability of GIHs in normal-weight adults over time. *Methods:* Plasma concentrations of ghrelin, leptin, GLP-1 (glucagon-like-peptide), and PP (pancreatic polypeptide) were assessed by enzyme-linked immunosorbent assay (ELISA) in 17 normal-weight, healthy adults in a longitudinal design at baseline and at follow-up six months later. The reliability of the measurements was estimated using intra-class correlation (ICC). In a second step, we considered the stability of GIH levels after controlling for changes in blood glucose and hemoglobin A1 (HbA1c) as well as self-reported physical activity and dietary habits. *Results:* We found excellent reliability for ghrelin, good reliability for GLP1 and PP, and moderate reliability for leptin. After considering glucose, HbA1c, physical activity, and dietary habits as co-variates, the reliability of ghrelin, GLP1, and PP did not change significantly; the reliability of leptin changed to poor reliability. *Conclusions:* The GIHs ghrelin, GLP1, and PP demonstrated good to excellent test–retest reliability in healthy individuals, a finding that was not modified after adjusting for glucose control, physical activity, or dietary habits. Leptin showed only moderate to poor reliability, which might be linked to weight fluctuations, albeit small, between baseline and follow-up assessment in our study sample. Together, these findings support that ghrelin, GLP1, and PP might be further examined as biomarkers in studies on weight control, with GLP1 and PP serving as anorexic markers and ghrelin as an orexigenic marker. Additional reliability studies in obese individuals are necessary to verify or refute our findings for this cohort.

## 1. Introduction

Gastrointestinal hormones (GIHs) are crucial for the regulation of a variety of physiological functions such as gastrointestinal (GI) secretion, digestion, and absorption of nutrients, energy and glucose homeostasis, and GI motility [1,2]. Moreover, GIHs have been linked to hunger, satiety, and appetite control as well as body weight control [3,4]. Considering the increasing number of overweight and obese children and adults worldwide, as well as associated severe comorbidities such as type 2 diabetes (T2D) and various cardiovascular diseases [5,6,7,8,9], a detailed understanding of the mechanisms underlying appetite regulation and body weight maintenance as well as the development of suitable interventions is important. As GIH levels can serve as biomarkers for beneficial GIH balance in the treatment of obesity and eating-related disorders and GIHs are determined in various studies investigating associated diseases, insight into the intra-individual variation of physiological levels of GIHs over time is essential to draw reliable conclusions. To enable sensitive monitoring of GIH changes induced by interventions between groups or in longitudinal comparisons, these GIH levels should be characterized by minor variability over time. Unfortunately, only limited data exist on the physiological distribution of GIH concentrations and on intra-individual variations of GIH levels in healthy non-obese individuals. So far, GIH levels were mainly measured in association with bariatric surgery [10,11,12,13,14,15], obesity [16], and T2D [17,18,19], either in a longitudinal design or one day after medication or supraphysiological GIH injections [17,18,20,21,22]. Most studies that examined healthy non-obese individuals had small sample sizes, and participants were often tested only once after ingestion or infusion of nutrients, GIHs, or their antagonists [23,24,25]. Studies with healthy individuals and repeated measurements of GIHs failed to reflect the physiological state of these hormones since participants received intraduodenal (ID) infusions of nutrients for subsequent measurements of plasma GI hormone and/or insulin concentrations [26,27]. Furthermore, to the best of our knowledge, no systematic assessment of GIH modulation by fasting serum parameters (e.g., glucose level and HbA1c) as well as dietary and physical activity habits has been conducted. Given that studies suggested that some GIHs might depend on the intensity of physical activity [28] and dietary habits [29], these parameters may have to be taken into consideration.

GI hormones have mostly been classified into two categories, i.e., orexigenic (appetite-stimulating, e.g., ghrelin) and anorexic (appetite-suppressing, e.g., leptin, GLP1, and PP). Ghrelin is largely produced by endocrine A-like cells in the gastric epithelium and is involved in GI motility, the secretion of stomach acid, glucose metabolism by inhibiting insulin secretion and controlling glycogenolysis and gluconeogenesis, and cardiovascular, immunologic, and reproductive processes [30,31]. Ghrelin triggers appetite; its serum levels increase, especially during fasting, and it initiates food consumption, leading to a positive energy balance with increased body weight. Leptin is an adipokine that is mainly produced by adipose tissue. It increases anorexigenic hormones and reduces orexigenic hormones in the hypothalamus, resulting in a reduced feeling of hunger or an increased feeling of satiety [32,33]. Moreover, the concentration of circulating leptin blood level is directly proportional to the fat level of the body and, thus, reflects its energy status [34]. GLP-1 (glucagon-like peptide 1) is a polypeptide that is synthesized by the neuroendocrine L-cells of the intestine. It is released during food intake and regulates glucose homeostasis by increasing glucose-stimulated insulin secretion and lowering hepatic glucose production [35]. In clinical trials, exogenous GLP-1 administration induced the feeling of satiety and reduced food intake, leading to its therapeutic implication in the treatment of T2D or obese patients [2,36]. Pancreatic polypeptide (PP) is a peptide hormone that is produced by the F-type cells of the pancreas [37]. PP levels in blood greatly increase after food consumption and remain elevated for several hours. The physiological relevance of PP is not fully understood, but it seems to inhibit pancreatic and gastric secretion as well as gastric motility [10,18].

Taken together, in-depth knowledge of the physiological degree of variation in GI hormones, using well-validated and accurate immunoassays that are comparable across studies, is currently not available. Such knowledge would be mandatory for interpreting changes in GI hormone levels (a) in pathological conditions such as obesity and its comorbidities and (b) after weight-reducing interventions. Additionally, the effects of physical activity and dietary habits on GIHs have not been systematically considered in these studies.

Therefore, the aim of our study is to determine intra-individual variation in the plasma concentrations of the four “classical” GIHs—ghrelin, glucagon-like peptide 1 (GLP1), pancreatic polypeptide (PP), and leptin [38]—in healthy normal-weight individuals without metabolic diseases. Plasma levels of GIHs were determined with a streptavidin-based ELISA in a longitudinal design at baseline and six months later. Additionally, we assessed the anthropometric parameters and fasting serum parameters of glucose and HbA1c, as well as of dietary habits and physical activity, to determine their impact on variability.

## 2. Material and Methods

### 2.1. Study Approval

The present study is a sub-group analysis of the study “*Neural correlates and mechanisms of cognitive changes induced by bariatric surgery for obesity per magna*” (registered at ClinicalTrials.gov: NCT01554228), which included obese participants undergoing bariatric surgery, an obese control group, and healthy normal or near-normal weight participants (current report). The protocol was approved by the local Ethics Committee of the University Medicine Greifswald (ethical approval code: BB/018/17b). The study was carried out in accordance with the principles of the Declaration of Helsinki. All participants provided written informed consent and received financial compensation for their participation in the study.

### 2.2. Participants

**Figure 1:**
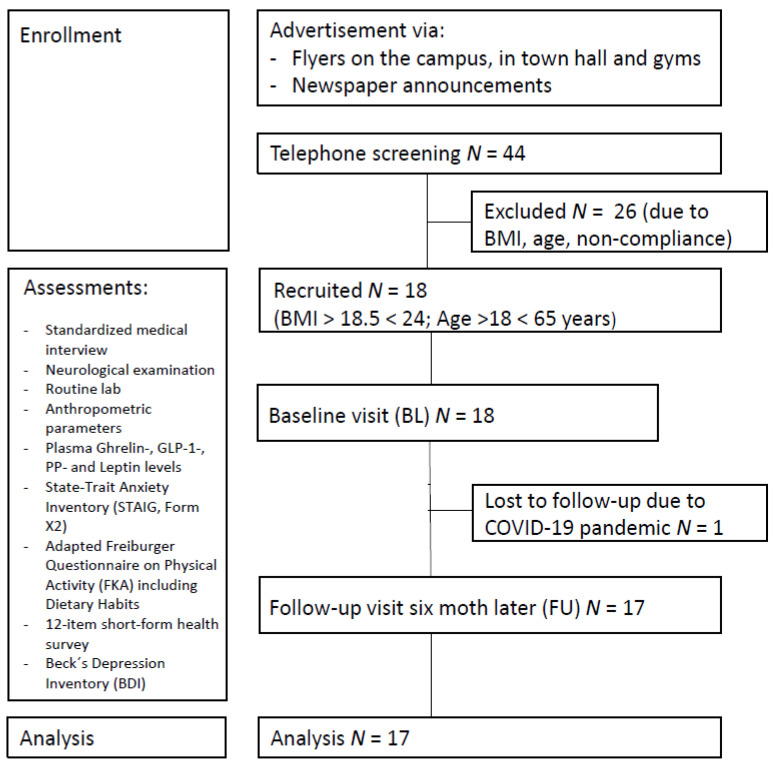
Study participant flowchart.

A total of 17 healthy normal or near-normal weight participants (mean BMI at first assessment: 21.97 ± 2.24 kg/m^2^, BMI range 17.96–26.23; 11 females; mean age: 37.7 ± 8.8 years; mean years of education: 16.5 ± 2.7 years) were included in the study. Recruitment took place between March 2019 and March 2020 via local newspaper announcements and flyers (see Figure 1). Exclusion criteria comprised severe untreated medical, neurological, and psychiatric conditions and not being a native German speaker, as all questionnaires were provided in German. None of the participants had a history of substance and alcohol abuse, diabetes, diseases affecting the gastrointestinal system, or dietary restrictions. The COVID-19 pandemic and subsequent economic shutdown led to delayed follow-up examinations, resulting in an average of 7.82 ± 2.81 months between baseline and follow-up. We had aimed to include a sample size of *N* = 40 in the control group in the overall ongoing trial, according to the protocol, and considered all available participants in July 2021 that met the inclusion criteria for the current analysis. For a summary of descriptive characteristics for all participants, see Table 1.

### 2.3. Study Design and Experimental Procedure

Participants were examined at two time points: baseline (BL) and follow-up (FU) after six months. After screening for eligibility via a telephone interview, potential participants received copies of the following questionnaires by mail prior to each examination: the State-Trait Anxiety Inventory (STAIG, Form X2, [39]), a 12-item short-form health survey (SF-12, [40]), the Freiburger Questionnaire on Physical Activity (FKA, [41]) and Beck’s Depression Inventory (BDI, [42]), to be completed before baseline and before follow-up.

For the on-site examination, the majority of participants arrived in the morning between 7:00 and 9:00 after an overnight fast for blood sampling. Due to scheduling difficulties (e.g., employment duties), some were examined in the afternoon. This applied to 8 participants (at baseline: *N* = 3, at follow-up: *N* = 3, at both time-points: *N* = 2). To determine whether blood collection at different times of the day affected fasting serum GIH levels for the 6 participants, permutation tests were performed for each GIH for this sub-group (see Supplementary Materials Tables S1 and S2, all *p*-values for each GIH > 0.05, respectively). Therefore, these participants were included in all analyses. Finally, we conducted a separate analysis excluding the 2 participants who were only examined in the afternoon (see Supplementary Materials Tables S3 and S4; for additional subgroup analysis please see also Supplementary Materials Table S5).

During each time-point, an MRI of the brain, a standardized medical interview, a neurological examination, a comprehensive neuropsychological test battery, and anthropometric parameters were assessed. MRI and neuropsychological measurements were not analyzed for the present sub-group analyses and will be reported elsewhere.

### 2.4. Physical Activity and Eating Habits

#### 2.4.1. Physical Activity Index

Similar to [43], we calculated an index of physical activity based on the Freiburger Questionnaire on Physical Activity (FKA, in German [41]). The FKA is a standardized questionnaire that includes questions about daily physical activity (e.g., climbing stairs, bicycling, or walking to work or shopping), leisure physical activity (e.g., garden work) and more athletic activities such as swimming, dancing, and tennis. Furthermore, to evaluate the specific energy consumption associated with each activity, we calculated the metabolic equivalent of task (MET) [44]. For this, the hours spent in a week on various activities, multiplied by their specific MET value, were summed and then divided by the square of body weight. For a summary of the above-described physical activities and their associated energy consumption for all participants, see Table 1.

#### 2.4.2. Dietary Habits Index

To assess participants’ eating habits, we calculated an additional index using an adapted version of a short, qualitative food frequency list employed in several large-scale German surveys [45]. This list included 37 questions about the frequency of consumption of various foods and dietary habits. Specifically, this list covers various forms of meat, poultry, fish, fruits, vegetables, grain products, dairy products, eggs, sweets, salty snacks, margarines, oils and other fat sources, and salt habits. In accordance with [43], participants were asked to rate their average food consumption for each item (i.e., frequency categories: ‘almost daily’, ‘several times per week’, ‘about once a week’, ‘several times per month’, ‘once a month or less’, or ‘never’). Finally, a dietary habits score was calculated, with a maximum score of 127 points corresponding to a healthy diet (e.g., a diet rich in raw fruits and vegetables, low-fat, whole-grain, and low-sugar products, fish, oils, and margarine high in unsaturated fats, moderately sheer meats, and reasonable salt habits) and a lower score corresponding to an unhealthy diet (e.g., a diet rich in fatty products, cured meats, soft drinks, sweets, butter, and salt, and low in fruits and vegetables).

### 2.5. Blood Sample Collection and Processing

Blood was sampled by venous puncture into BD vacutainer blood collection tubes: Na_3_-Citrat tubes for the determination of blood coagulation parameters and fasting glucose; serum SST II Advance tubes for the determination of C-reactive protein (high sensitivity CRP assay), creatinine, estimated glomerular filtration rate (eGFR), GGT, total cholesterol, triglyceride, and the high-to-low density lipoprotein (HDL-to-LDL) ratio; K2-EDTA tubes for the determination of small blood count parameters and glycated hemoglobin A1c (HbA1c) (pre-cooled and room temperature) or BD^TM^ P800 for the standardized determination of glucagon-like peptide-1 (GLP-1), pancreatic polypeptide (PP), glucagon, and ghrelin. BD^TM^ P800 tubes contain a protease- and esterase cocktail to stabilize peptides. The sequence of tubes was defined during blood withdrawal and comprised Na_3_-Citrat, serum, K2-EDTA, and BD^TM^ P800. After the centrifugation of serum SST II Advance tubes (3000× *g*, 5 min, 18–25 °C) and BD^TM^ P800 tubes (1300× *g*, 20 min, 18–25 °C), the aliquots were immediately stored at −80 °C until analysis. Small blood count parameters and serum levels of C-reactive protein (high sensitivity CRP assay), creatinine, the estimated glomerular filtration rate (eGFR), GGT, fasting glucose, glycated hemoglobin A1c (HbA1c), total cholesterol, triglyceride, and the high-to-low density lipoprotein (HDL-to-LDL) ratio were analyzed by the Department of Clinical Chemistry at the University Medicine Greifswald immediately after sampling. For a summary of all fasting serum parameters, see Table 2.

### 2.6. Laboratory Analysis

**Figure 2:**
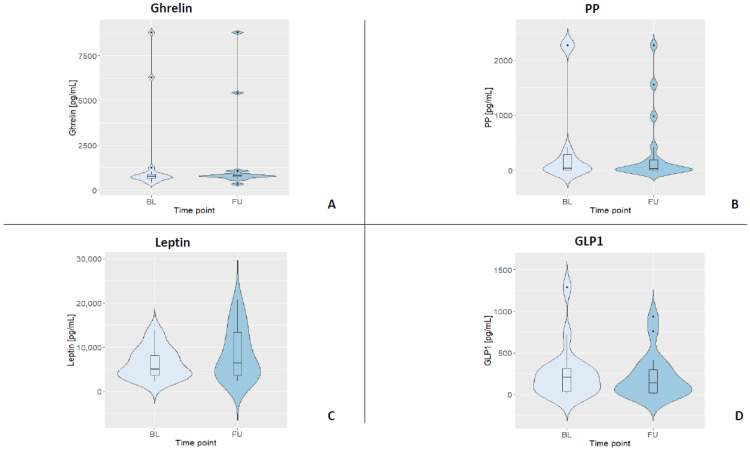
Violin plots displaying the kernel distribution of all GIH levels for both assessments (BL = baseline, FU = follow-up). Box plots include the first through third quartiles, with the horizontal line representing the median. Black dots represent outliers. (**A**) Distribution of ghrelin. (**B**) Distribution of PP. (**C**) Distribution of leptin. (**D**) Distribution of GLP1.

To determine the plasma concentration of gastrointestinal hormones, ELISAs (enzyme-linked immunosorbent assays; ThermoFisher Scientific, Vienna, Austria) for human ghrelin-, GLP-1-, PP-, and leptin were performed. For all kits, the manufacturer stated an intra-assay CV < 10% and an inter-assay CV < 12%, respectively. This performance was reached for three out of the four GIHs, while GLP-1 had an intra-assay variance of 13.8% in our hands. All ELISAs were strictly carried out according to the manufacturer’s instructions and on plates with the same batch number by the same trained staff member. All standard curves were based on duplicates. Samples were diluted in assay buffer for analyses (ghrelin 1:10, leptin 1:50, GLP-1 1:6, and PP 1:2). Samples below the detection limit were set to 0, and samples above the highest standard were set to 2276.02 pg/mL (i.e., for PP). All samples were analyzed in duplicates. For a summary of all fasting GIH levels and their distributions, see Table 3 and Figure 2.

### 2.7. Statistical Analysis

Statistical analyses were performed with R [46]. Violin plots were created using the R package ggplot2 [47]. Longitudinal plots were created using standard R package stats [48]. A forest plot was created using the R package forestplot [49]. Results were considered statistically significant at a *p*-value of <0.05. All statistical analyses performed are described in detail below.

#### 2.7.1. Permutation Test for Paired Data

To compare the mean values of GI hormones and fasting serum parameters between baseline and follow-up, a permutation test for paired data was performed for each parameter. Permutation tests are robust non-parametric test procedures known to accurately control Type I errors (rejecting a true null hypothesis) even for very small sample sizes [50]. Based on a Monte Carlo estimate of the *p*-value, we hypothesized that each parameter (i.e., GIHs and fasting serum parameters) would show no difference between the two assessments. Under this hypothesis, the paired data points were considered to be equally flexible, on average, for each participant (i.e., a participant’s baseline GLP1 value could also be the follow-up value on average and vice versa). Within each participant, assessment values were randomly permuted. Then, after each permutation, the mean values were calculated by assessment for each participant. This process was repeated for 100,000 iterations. The test level was two-sided and set to 0.05.

#### 2.7.2. Intra-Class Correlation (ICC)

ICC is a statistical method to estimate the reliability of ratings or measurements for clustered data. According to [51], the following cut-off values and interpretations can be made: ICC < 0.5 means poor reliability, ICC 0.5–0.75 means moderate reliability, ICC 0.75–0.9 means good reliability, and ICC > 0.90 means excellent reliability. First, ICC was calculated for each GIH using the ‘icc’ function of the R package irr [52]. In accordance with our study design, we chose a two-way mixed-effects model based on a single measurement and absolute agreement as the reliability measure ICC (A,1) [53]. Furthermore, we considered changes in GIH levels driven by variations of glucose (mmol/L) and HbA1c (%) parameters as well as physical activity (h/week) and dietary habits index. For this, we calculated an adjusted ICC by using the estimated variance components of the linear mixed-effects model, with the above-mentioned co-variates as fixed effects and a random intercept to account for participants’ individual differences by considering data from the two assessments.

## 3. Results

Descriptive data of all participants are shown in Table 1. All parameters of small blood count, CRP, creatinine, eGRF, and GGT were within their specific reference range (see Table S1 for reference ranges), indicating that all participants were not suffering from acute illness. During each assessment, the majority of participants subjectively rated their physical activity compared to their peers as equally active, more active, or much more active (BL: 88%, FU: 82%). Additionally, most participants rated their physical condition as fair, good, or very good during both assessments (BL: 76%, FU: 88%). Eleven participants (around 65%) indicated that they had never smoked, five participants were previous smokers (29%; M = 5 cigarettes per day ± 2.29), and one participant was a smoker (6%; 7 cigarettes per day) during baseline assessment. The dietary habit index revealed an average value of 83.35 ± 7.84 points (BL) and 84.06 ± 6.36 (FU), with 127 points being the maximum score corresponding to a healthy diet. In sum, baseline parameters, including physical activity and dietary habits, remained stable for the participants over the course of this study. 

### 3.1. Fasting Serum Parameters

Table 2 depicts the mean values and standard deviations of the fasting serum parameters for all participants and for each assessment. Again, all parameters were within their reference range (see Table S1), indicating that participants showed physiological values for these standard serum parameters. Permutation tests for each serum parameter revealed no significant differences between baseline and follow-up assessment (all *p*-values > 0.05).

### 3.2. Fasting GIH Levels

**Figure 3:**
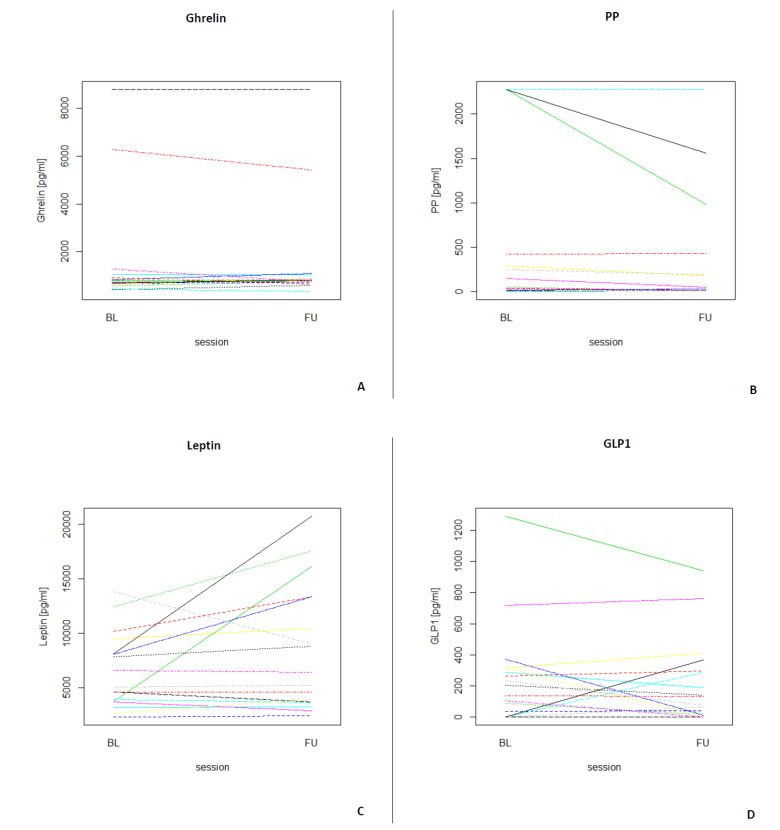
Longitudinal plots of the different GIHs for each participant (*N* = 17) for both assessments (BL = baseline, FU = follow-up). (**A**) Individual ghrelin values for all participants. (**B**) Individual PP values for all participants. (**C**) Individual leptin values for all participants. (**D**) Individual GLP1 values for all participants.

Table 3 depicts the median and interquartile ranges (1st quartile and 3rd quartile) of the four fasting GIH levels for all participants and for each assessment. Figure 3 shows the individual values of GIHs of all participants for both assessments. For a visualization of the individual GIH values divided by fasting and non-fasting status, see Figures S1–S4. Permutation tests for each GIH level revealed no significant differences between baseline and follow-up assessments for ghrelin, leptin, and GLP1 (all *p*-values > 0.05), while the permutation test for PP showed a significant difference between baseline and follow-up (*p* < 0.05). After excluding the two participants who were only assessed in the afternoon, the results of the permutation test did not show a different pattern (see Table S2; ghrelin, leptin, and GLP1, all *p*-values > 0.05; PP *p* < 0.01).

### 3.3. ICC

The ICC coefficient was computed to assess the reliability of fasting GIH levels in 17 participants between the two assessments. Table 4 shows that reliability for ghrelin (kappa = 0.99, *p* < 0.001) was excellent. The ICC coefficient for PP (kappa = 0.89, *p* < 0.001) and GLP1 (kappa = 0.79, *p* < 0.001) showed good reliability between both assessments, while the ICC coefficient for leptin (kappa = 0.51, *p* < 0.05) revealed moderate reliability. To evaluate whether GIH values of participants who were only assessed in the afternoon substantially affected results, we excluded these two participants and computed again the ICC coefficient for each GIH level (see Table S3). The results show no substantial changes in reliability for the three hormones (i.e., ghrelin excellent reliability, kappa = 0.99, *p* < 0.001; PP good reliability, kappa = 0.89, *p* < 0.001; and GLP-1 good reliability, kappa = 0.82, *p* < 0.001). Only the reliability of leptin changed substantially from moderate to poor reliability (kappa = 0.48, *p* < 0.05).

**Figure 4:**
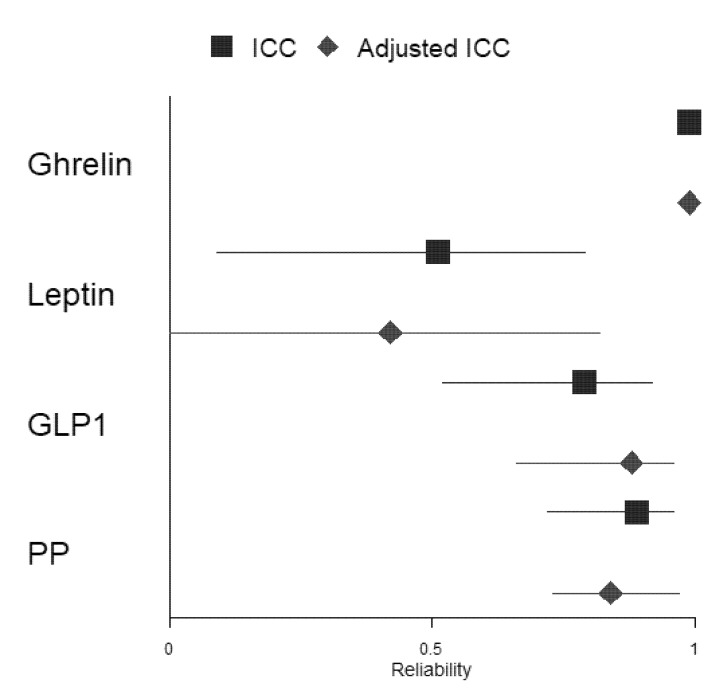
Forest plot depicting ICCs and adjusted ICCs with their respective 95% confidence intervals for all included GIHs.

In a second step, we included fasting serum parameters glucose and HbA1c as well as total physical activity and dietary habits indices for all assessments as fixed effects in the mixed-effects model. Adjusted ICC coefficients for ghrelin, GLP1, and PP did not change substantially compared to ICC coefficients, including only ghrelin, GLP1, or PP levels when considering all participants, respectively (ghrelin: both ICC coefficients with excellent reliability; GLP1 and PP: both ICC coefficients with good reliability). However, the adjusted ICC coefficient for leptin changed substantially from moderate to poor reliability compared to the ICC coefficient, including the only-leptin level (for a forest plot of the ICC and adjusted ICC, see Figure 4). Excluding the two participants who were assessed in the afternoon only changed the adjusted ICC of leptin from moderate to poor (see Table S3).

## 4. Discussion

The aim of the present study was to evaluate the intra-individual variability of plasma concentrations of the four GI hormones *ghrelin*, *GLP-1*, *PP*, and *leptin* in healthy adults; by repeated measurements from blood samples obtained at baseline and after 6 months. We reached an intra-assay CV of <10% for ghrelin, leptin, and PP, while GLP-1 had an intra-assay CV of 13.8% in our hands. This demonstrates that the ghrelin, leptin, and PP assays were well performed; our resulting respective GIH values were accurate, although the performance of GLP-1 was slightly above the acceptable range.

Moreover, we were interested in the influence of physical activity and dietary habits as well as HbA1c and glucose levels on the stability of these hormones over time. To the best of our knowledge, this is the first study addressing intra-individual variability of these four key GI hormones in a healthy cohort. However, because of our small sample size, this study should be considered a pilot study. Since GI hormones can be used, for example, in longitudinal cohorts as well as interventional studies on obesity, T2D, and eating disorders [2,54,55], a better understanding of variations of GI hormones in healthy adults could help to distinguish physiological from pathological variability in individuals with obesity and/or eating disorders and help us to decide which hormones might be used as sensitive biomarkers in interventional studies. In this context, the ICC can be a useful tool as it provides insights into the test–retest reliability of repeated measurements and, thus, helps to evaluate the individual and measurement variability of biomarkers [51,56].

The results of this pilot study indicate that ghrelin, PP and GLP1 displayed good to excellent reliability, while leptin showed only moderate reliability. The ICC coefficient for the orexigenic hormone ghrelin was the only GIH that revealed excellent reliability over time. This is in line with a previous study that showed stable ghrelin levels in healthy patients and also cancer patients over time (i.e., 5 years) [57]. The ICC coefficients in previous studies were comparable between studies, indicating that ghrelin seems to have good reliability across different pathological and non-pathological populations [57,58]. However, compared with our results, the reliability of ghrelin in these studies was lower, possibly because we utilized the stabilizing effect of protease, esterase, and DPP IV inhibitors during blood sample processing.

ICC coefficients of the anorexic hormones GLP1 and PP revealed good reliability over time. Since there is no general agreement on the interpretation of ICC and given the high variability of blood level values, good reliability for these two GI hormones seems to be tolerable. Studies on the reliability of GLP1 levels evaluated with ICC are sparse. For instance, Nair et al. [59] demonstrated that the reliability of plasma secretion of GLP1 was >0.80 between three assessments. However, we could not find any study investigating the reliability of PP levels over time. Therefore, our study provides the first evidence that PP levels are reliable and stable over a period of 6 months.

ICC for the anorexic hormone leptin revealed moderate reliability over a period of 6 months. Previous studies have shown mixed results on the reliability of leptin levels in different pathological cohorts. For instance, Stattin et al. [60] showed that the ICC coefficient for leptin over a period of one year in colon and rectum cancer patients was high. In a study with obese participants, leptin levels showed good reliability [61]. However, when examining differences in reliability between male and female participants, the reliability for female participants was moderate, whereas the reliability for male participants was good. Since our sample consisted of 11 females and only 6 males, the moderate reliability of leptin over the entire sample could be attributed mainly to lower reliability in the female group. However, a comparison of gender groups, albeit with small group sizes, particularly in the male group, did not indicate that the ICC in males was higher. However, since the leptin level is strongly correlated with body fat and most of the evidence of the reliability of leptin is based on obese participants, gender differences might not be evident for healthy non-obese participants, an issue that should be further explored in larger samples. Another possible explanation for the moderate reliability of leptin in our study could be due to the weight variability between baseline and follow-up within participants. Permutation tests for weight and BMI revealed significant differences for both parameters (*p* < 0.05 and *p* < 0.001) in our study sample. Since leptin seems to be sensitive to changes in body fat due to weight gain or loss, fluctuations in leptin between time points and the corresponding low test–retest reliability could be explained by weight fluctuations in individual subjects [62,63]. Indeed, an exploratory analysis revealed that the ICC coefficient for participants with (small) weight changes between baseline and follow-up showed poor reliability (kappa = 0.29, *p* > 0.05), whereas participants with no weight changes showed good test–retest reliability (kappa = 0.86, *p* < 0.01). Nevertheless, this result should be handled carefully since our sample size is small, and the ICC of participants with weight changes was not significant. However, taking these results into consideration, leptin could also serve as a potential biomarker, especially in studies of weight control, since it seems to be highly sensitive to weight changes.

Moreover, we were interested in the stability of reliability after considering glucose and HbA1c parameters as well as physical activity and dietary habits as co-variates. ICC coefficients did not change substantially for ghrelin, PP, and GLP1 but changed from moderate to poor reliability for leptin. Thus, at least in non-obese individuals, day-to-day variation in eating habits, physical activity, and parameters of glucose control might not explain possible changes in longitudinal or interventional studies over time for three of the four GI hormones. The reliability of leptin measurements, which was initially only moderate, was further degraded by the inclusion of (minor) variations in dietary habits and corresponding glucose/HbA1c levels and physical activity. Again, this could be attributed to the weight variability between baseline and follow-up assessments in our study sample, which should also be reflected in higher variability, at least for dietary habits and physical activity between both assessments. Interestingly, our study provides a first hint that the time point of blood collection (i.e., in the morning or evening) and, therefore, the effect of fasting state on the assessed GI hormones might not be as substantial as expected, which could improve the feasibility of longitudinal or interventional studies using GIHs as biomarkers. However, because our sample size was small (*N* = 9 with over-night fasting status vs. *N* = 6 only over-night fasting status during one assessment time point vs. *N* = 2 without over-night fasting status), we could not draw any reliable conclusion, and, therefore, this result should be handled with caution, particularly since there is ample evidence in the literature that GIH concentrations can change depending on fasting status ([64,65,66]; but see [64] for an insignificant difference between fasting and non-fasting leptin plasma levels in healthy controls).

Taken together, the choice of the most reliable parameters is essential for the quality and outcome of studies investigating GIH levels as biomarkers over time or between groups. Our study provides evidence for the test–retest reliability of GIH hormones by showing that the hormones ghrelin, GLP1, and PP were (i) stable over a period of 6 months when compared to baseline measurements and that (ii) their stability was not affected by dietary habits, physical activity, and blood glucose levels. Our findings will enable researchers to adapt their future study designs accordingly.

When considering the results of the present study, the following limitations should be taken into account. In our study, a small cohort was evaluated. However, our participants were well-characterized (i.e., fasting serum parameters, dietary and lifestyle habits), and our study size was comparable to similar studies [26,27]. Future studies should include a more gender-balanced design to determine the influence of gender on the reliability of GIH. Furthermore, since the participants were healthy and showed only small variations over time in the assessed co-variates (i.e., the physical activity and dietary habits indices), the results may not be applicable to obese populations, an issue that should also be addressed in future studies. Finally, due to scheduling difficulties in several participants, we were not able to always collect blood samples in the morning; thus, several blood draws were not conducted under fasting conditions. Interestingly, the time point of blood collection and fasting state of blood levels did not affect stability significantly, thus providing the first indication that the level of GIH is independent of fasting status. Nevertheless, because of its limitations (i.e., small sample size and not all blood draws in the morning), this study should be considered a pilot study, and results should be viewed with caution.

## 5. Conclusions

Assessing variability and reliability of GI hormones over time, using well-validated and accurate immunoassays that are comparable across studies, is crucial to understanding and estimating longitudinal physiological and non-physiological changes and to optimally designing future interventional studies to reduce blood draws to a minimum.

Our findings in this pilot study show good to excellent test–retest reliability in near-normal weight participants for ghrelin, GLP1, and PP. Therefore, these GIHs might be further examined as biomarkers in studies on weight control. Leptin showed only moderate to poor reliability, which would rather discourage its examination as a biomarker in longitudinal cohort or intervention studies. However, an exploratory analysis suggested that even small weight fluctuations might affect leptin reliability, which might be a hint that leptin could indeed be included as a sensitive biomarker for weight changes in future studies. Additional reliability studies should be conducted on obese individuals to verify or refute our findings for this cohort.

## Figures and Tables

**Table 1:** Descriptive characteristics of participants at each assessment.

Variable	*N*	Baseline	Follow-Up	*p*
**Small blood count and CRP**				
Red blood cells (Tpt/L)	17	4.70 (4.1, 5.00) ^c^	4.70 (4.5, 4.8) ^c^	0.99
White blood cells (Gpt/L)	17	5.43 (4.89, 6.56) ^c^	4.81 (4.39, 6.27) ^c^	0.17
Platelets (Gpt/L)	17	241.00 (202.00, 267.00) ^c^	233.00 (217.00, 274.00) ^c^	0.32
Hemoglobin (mmol/L)	17	8.50 (7.6, 8.9) ^c^	8.30 (7.9, 8.7) ^c^	0.99
Hematocrit	17	0.40 (0.37, 0.43) ^c^	0.40 (0.38, 0.42)	0.73
MCH (fmol)	17	1.78 (1.75, 1.84) ^c^	1.78 (1.74, 1.88)^c^	0.99
MCV (fl)	17	87.50 (84.60, 89.30) ^c^	85.90 (83.6, 88.80) ^c^	0.50
MCHC (mmol/L)	17	20.40 (20.10, 21.00) ^c^	20.80 (20.40, 21.20) ^c^	0.28
RDW (%)	17	12.60 (12.00, 14.70) ^c^	12.20 (11.90, 14.60) ^c^	0.73
CRP (mg/L) ^a^	17	0.35 (0.24, 0.79) ^c^	0.50 (0.21, 1.01) ^c^	0.91
**Kidney and Liver Function**				
Creatinine	17	73.00 (61.00, 80.00) ^c^	70.00 (61.00, 83.00) ^c^	0.72
eGRF	17	>60	>60	-
GGT	17	0.36 (0.35, 0.41) ^c^	0.35 (0.31, 0.44) ^c^	0.59
**Diet**				
Dietary habits index	17	82.00 (79.00, 88.00) ^c^	83.00 (81.00, 90.00) ^c^	0.66
Alcohol consumption (drinks/last 3 days) ^d^	17	1.33 (0, 3.17) ^c^	1.67 (0, 3.50) ^c^	0.72
**Lifestyle**				
Daily physical activity (h/week)	17	1.54 (0.03, 2.81) ^c^	1.34 (0.17, 5.53) ^c^	0.20
Leisure physical activity (h/week)	17	4.00 (1.00, 9.00) ^c^	5.67 (2.00, 8.00) ^c^	0.61
Athletic physical activity (h/week)	17	2.00 (0.63, 5.50) ^c^	2.00 (0.00, 3.50) ^c^	0.82
Total physical activity index (h/week)	17	11.54 (4.57, 16.70) ^c^	10.53 (8.53, 13.52) ^c^	0.68
Energy consumption daily physical activity (kcal/week)	16 ^b^	165.29 (19.48, 430.22) ^c^	251.99 (64.70, 1364.77) ^c^	0.25
Energy consumption leisure activity (kcal/week)	16 ^b^	252.00 (87.13, 723.63) ^c^	371.50 (122.63, 730.50) ^c^	0.93
Energy consumption athletic physical activity (kcal/week)	16 ^b^	866.50 (408.63, 1506.50) ^c^	710.50 (68.00, 1073.25) ^c^	0.74
Energy consumption total physical activity (kcal/week)	16 ^b^	1475.88 (687.40, 3200.02) ^c^	1583.27 (933.06, 3855.55) ^c^	0.51
Weight (kg)	16 ^b^	67.00 (57.00, 76.25) ^c^	68.00 (59.25, 76.25) ^c^	<0.05 *
BMI (kg/m^2^)	16 ^b^	21.18 (20.46, 23.08) ^c^	22.44 (21.19, 23.77) ^c^	<0.01 **
Sleep duration (h/week)	17	49.00 (42.00, 56.00) ^c^	52.50 (49.00, 56.00) ^c^	0.27

^a^ Values below and above the detection level were set to 0 and 0.165, respectively. ^b^ One participant was excluded due to missing weight at follow-up assessment. ^c^ Values are presented as median and interquartile range. ^d^ Alcohol consumption was calculated, including the last weekend (Saturday and Sunday) and one weekday (excluding Fridays) prior to assessment. Abbreviations: MCH, mean corpuscular hemoglobin; MCV, mean corpuscular volume; MCHC, mean corpuscular hemoglobin concentration, RDW, red cell distribution; eGRF, estimated glomerular filtration rate; GGT, gamma-glutamyl transferase; BMI, body mass index. * *p* < 0.05, ** *p* < 0.01.

**Table 2:** Fasting serum parameters (*N* = 17).

**Serum Parameter**	**BL**	**FU**	***p***
Glucose (mmol/L)	4.90 ± 0.66	5.22 ± 0.54	0.123
HbA1c (%)	5.37 ± 0.19	5.41 ± 0.26	0.501
Total cholesterol (mmol/L)	4.79 ± 0.81	5.02 ± 1.11	0.212
Triglyceride (mmol/L)	1.11 ± 0.51	1.08 ± 0.44	0.604
LDL/HDL Ratio ^a^	1.55 ± 0.46	1.66 ± 0.46	0.077

Values are presented as means ± SD. Abbreviations: HbA1c, hemoglobin A1c; LDL, low-density lipoprotein; HDL, high-density lipoprotein. ^a^ LDL/HDL ratio was calculated as LDL-Cholesterol/ HDL-Cholesterol.

**Table 3:** Fasting GIH levels (*N* = 17).

**GIH Levels**	**BL**	**FU**	***p***
Ghrelin (pg/mL)	771.92 (662.63, 898.21)	804.76 (751.65, 867.29)	0.719
Leptin (pg/mL)	5056.90 (3704.31, 8132.15)	6407.82 (3639.22, 13,352.38)	0.076
GLP1 (pg/mL)	168.78 (203.21, 315.32)	134.00 (15.07, 294.95)	0.438
PP (pg/mL)	37.29 (12.49, 289.28)	27.47 (9.37, 188.52)	0.011

Values are presented as median and interquartile ranges.

**Table 4:** ICC(A,1) and adjusted ICC for all fasting GIH levels (all *N* = 17).

GIH Levels	ICC	95% CI_ICC_	Adjusted ICC	95% CI_adICC_
Ghrelin	0.99	0.98, 1	0.99	0.98, 1
Leptin	0.51	0.09, 0.79	0.42	0, 0.82
GLP1	0.79	0.52, 0.92	0.88	0.66, 0.96
PP	0.89	0.72, 0.96	0.84	0.73, 0.97

95% CI with lower and upper boundaries. Adjusted ICC was calculated by using the variance components of a linear mixed-effects model with the following fixed effects: glucose, HbA1c, total physical activity, and dietary habits index; a random intercept was used to account for the participants’ individual differences. Abbreviations: ICC, intra-class correlation; CI_ICC_, confidence interval of ICC; CI_adICC_, confidence interval of adjusted ICC.

## Data Availability

The datasets used and analyzed in the current study are available from the corresponding author on reasonable request.

## Supplementary Materials

The following Supplementary Materials are available online at https://www.mdpi.com/article/10.3390/nu13113809/s1, Table S1: Fasting GIH Levels for participants with blood drawing either in the morning or in the afternoon; Table S2: Fasting serum parameters for participants with blood drawing either in the morning or in the afternoon; Table S3: Fasting GIH Levels (N = 15); Table S4: ICC(A,1) and adjusted ICC for all fasting GIH levels (N = 15); Table S5: Fasting GIH Levels for participants with blood drawing only in the morning and blood drawing only in the afternoon; Table S6: Reference ranges for variables of small blood count and CRP, variables of kidney and liver function, and fasting serum parameters; Figure S1: Longitudinal plots of Ghrelin for each participant for both assessments divided by fasting status; Figure S2: Longitudinal plots of PP for each participant for both assessments divided by fasting status; Figure S3: Longitudinal plots of Leptin for each participant for both assessments divided by fasting status; Figure S4: Longitudinal plots of GLP1 for each participant for both assessments divided by fasting status.
