# Supplementary material for: Gastrointestinal Hormones in Healthy Adults: Reliability of Repeated Assessments and Interrelations with Eating Habits and Physical Activity
Source: Nutrients. 2021 Oct 26;13(11):3809. doi: 10.3390/nu13113809 (PMC8624073; doi:10.3390/nu13113809)
Supplement: Supplementary file 1 [file nutrients-13-03809-s001.zip › nutrients-1406994-supplementary.pdf]

Table S1. Fasting GIH Levels for participants with blood drawing either in the morning or in the afternoon ($N = 6$).

GIH Levels	BL	FU	*p*
Ghrelin [pg/ml]	650.70 (488.82, 761.35)	854.31 (656.13, 866.48)	0.09
Leptin [pg/ml]	7941.88 (4887.21, 9121.07)	8919.14 (4911.55, 10092.20)	0.75
GLP1 [pg/ml]	274.10 (210.65, 357.70)	214.03 (93.20, 381.04)	0.78
PP [pg/ml]	195.28 (64.34, 278.22)	111.68 (32.80, 185.04)	0.12

Values are presented as median and interquartile range.

Table S2: Fasting serum parameters for participants with blood drawing either in the morning or in the afternoon ($N = 6$)

Serum Parameters	BL	FU	*p*
Glucose [mmol/l]	4.60 ± 0.52	5.22 ± 0.55	0.12
HbA1c [%]	5.33 ± 0.14	5.28 ± 0.27	0.62
Total cholesterol [mmol/l]	4.73 ± 0.42	5.23 ± 1.24	0.31
Triglyceride [mmol/l]	1.01 ± 0.40	0.91 ± 0.37	0.75
LDL/HDL Ratio[a]	1.49 ± 0.36	1.61 ± 0.44	0.18

Values are presented as means ± SD. Abbreviations: HbA1c, Hemoglobin A1c; LDL, low-density lipoprotein; HDL, high-density lipoprotein. [a] LDL/HDL Ratio was calculated as LDL-Cholesterol/ HDL-Cholesterol.

Table S3: Fasting GIH Levels ($N = 15$)

GIH Levels	BL	FU	*p*
Ghrelin [pg/ml]	771.92 (650.70, 962.92)	806.39 (743.52, 956.31)	0.757

Leptin [pg/ml]	6587.61 (3802.92, 8806.87)	8789.21 (3629.11, 13365.95)	0.097
GLP1 [pg/ml]	203.21 (63.41, 301.98)	139.60 (28.36, 331.55)	0.618
PP [pg/ml]	46.53 (14.68, 355.27)	27.47 (9.55, 306.60)	< 0.01**

Values are presented as median and interquartile range. * $p < 0.05$, ** $p < 0.01$, *** $p < 0.001$.

Table S4: ICC(A,1) and adjusted ICC for all fasting GIH levels (all $N = 15$)

GIH Levels	ICC	95% CI_{ICC}	adjusted ICC	95% CI_{adICC}
Ghrelin	0.99	0.98, 1	0.99	0.98, 1
Leptin	0.48	0.02, 0.78	0.40	0, 0.85
GLP1	0.82	0.54, 0.93	0.87	0.68, 0.97
PP	0.89	0.70, 0.96	0.85	0.71, 0.97

95% CI with lower and upper bound. Adjusted ICC was calculated by using the variance components of a linear-mixed effects model with the following fixed effects: glucose, HbA1c, total physical activity and dietary habits index. Abbreviations: ICC, intra class correlation; CI_{ICC}, confidence interval of ICC; CI_{adICC}, confidence interval of adjusted ICC.

Table S5: Fasting GIH Levels for participants with blood drawing only in the morning ($N = 9$) and blood drawing only in the afternoon ($N = 2$)

GIH Levels	BL Morning Blood Draw	BL Afternoon Blood Draw
Ghrelin [pg/ml]	898,21 (694.52, 1261.39)	796,62 (756.53, 836.71)

Leptin [pg/ml]	4590,63 (3704.30, 8132.50)	3839,44 (3230.71, 4448.17)
GLP1 [pg/ml]	104.28 (36.76, 264.86)	159,76 (81.03, 238.49)
PP [pg/ml]	31,61 (4.00, 421.26)	12,82 (8.41, 17.24)
	FU Morning Blood Draw	FU Afternoon Blood Draw
Ghrelin [pg/ml]	804,75 (785.01, 1045.33)	755,42 (753.53, 757.31)
Leptin	6407,82 (3639.22, 16147.79)	4550,40 (4211.09, 4889.70)
GLP1 [pg/ml]	134,00 (15.07, 294,94)	32,64 (17.47, 47.81)
PP [pg/ml]	9,72 (4.77, 424.67)	17,04 (11.29, 22.78)

Values are presented as median and interquartile range.

Table S6: Reference ranges for variables of small blood count and CRP, variables of kidney and liver function, and fasting serum parameters

Variable	Reference Range Male	Reference Range Female
**Small blood count and CRP**		
Red blood cells [Tpt/l]	4.6 – 6.2	4.2 – 5.4
White blood cells [Gpt/l]	4.3 – 10.0	4.3 – 10.0
Platelets [Gpt/l]	140 – 440	140 – 440
Hemoglobin [mmol/l]	8.6 – 11.2	7.4 – 10.0
Hematocrit	0.40 – 0.51	0.35 – 0.47
MCH [fmol]	1.68 – 2.00	1.68 – 2.00

MCV [fl]	80 – 95	80 – 95
MCHC [mmol/l]	18.5 – 22.5	18.5 – 22.5
RDW [%]	11.0 – 16.0	11.0 – 16.0
hs-CRP [mg/l]	< 5.0	< 5.0
**Kidney and liver function**		
Creatinine	49 – 97	42 – 80
eGRF	> 60	> 60
GGT	0 – 0.96	0 – 0.65
**Fasting serum parameters**		
Glucose [mmol/l]	3.9 – 6.4	3.9 – 6.4
HbA1c [%]	< 6.5	< 6.5
Total cholesterol [mmol/l]	< 6.0	< 6.0
Triglyceride [mmol/l]	0 – 1.9	0 – 1.9
LDL/HDL Ratio	< 3	< 3

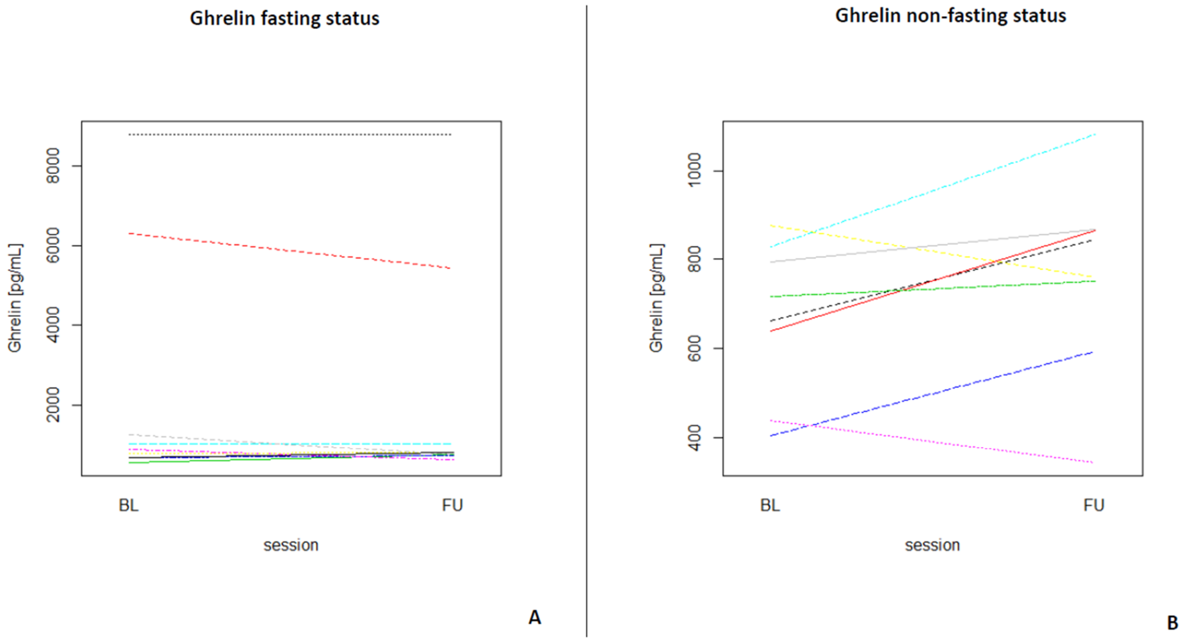

Fig. S1: Longitudinal plots of Ghrelin for each participant for both assessments (BL = baseline, FU = follow-up) divided by fasting status. **(A)** Longitudinal plot for participants with over-night fasting status (only assessed in the morning, $N = 9$). **(B)** Longitudinal plot for participants without over-night fasting status (assessed in the afternoon during BL or FU or both assessments, $N = 8$).

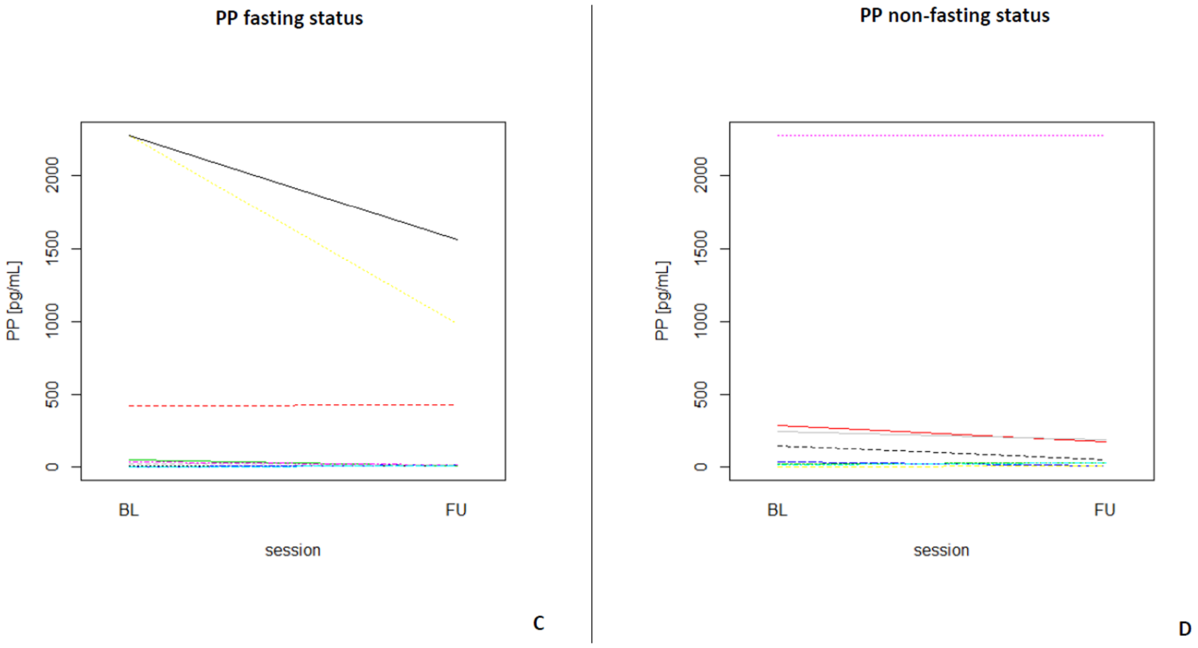

Fig. S2: Longitudinal plots of PP for each participant for both assessments (BL = baseline, FU = follow-up) divided by fasting status. **(C)** Longitudinal plot for participants with over-night fasting status (only assessed in the morning, $N = 9$). **(D)** Longitudinal plot for participants without over-night fasting status (assessed in the afternoon during BL or FU or both assessments, $N = 8$).

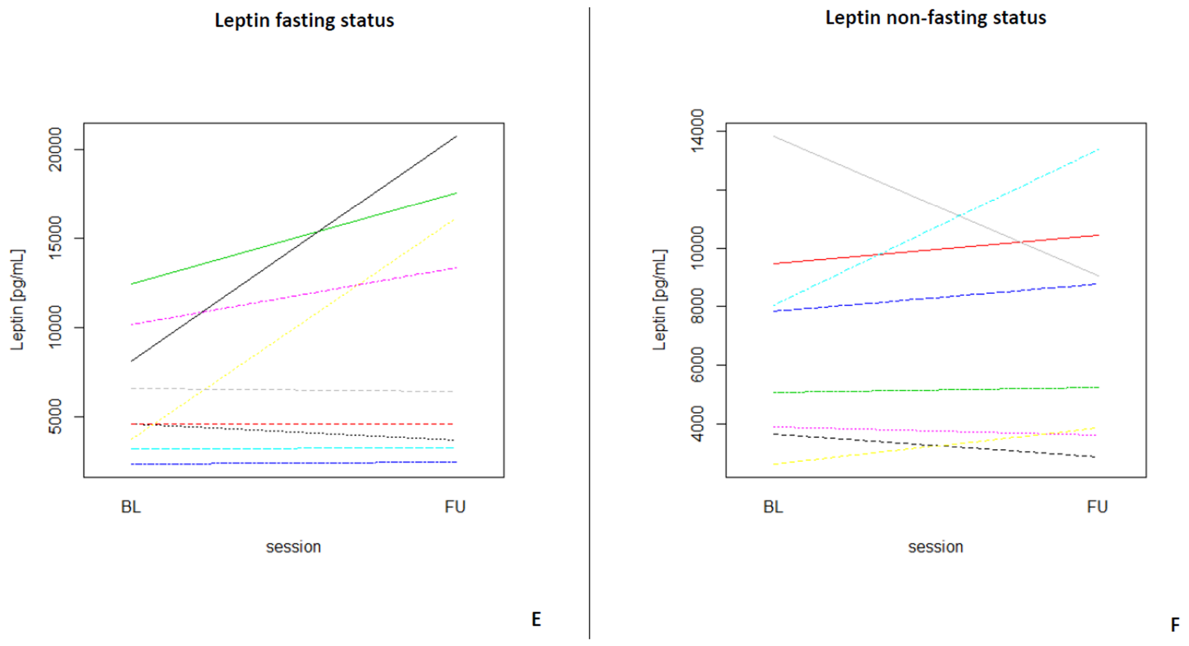

Fig. S3: Longitudinal plots of Leptin for each participant for both assessments (BL = baseline, FU = follow-up) divided by fasting status. **(E)** Longitudinal plot for participants with over-night fasting status (only assessed in the morning, *N* = 9). **(F)** Longitudinal plot for participants without over-night fasting status (assessed in the afternoon during BL or FU or both assessments, *N* = 8).

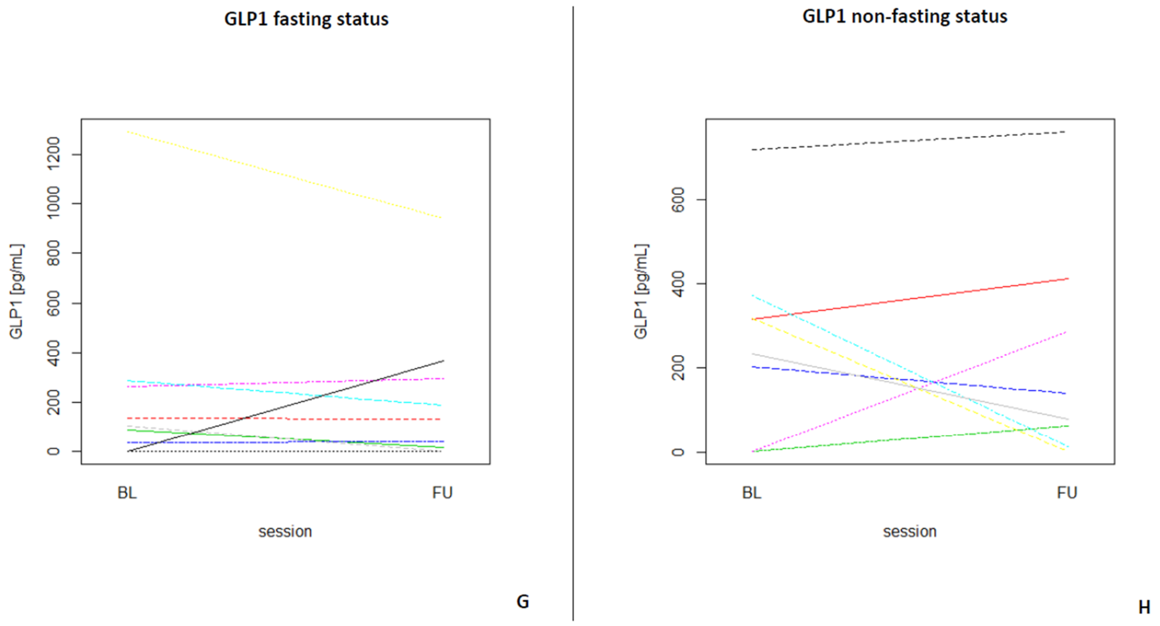

Fig. S4: Longitudinal plots of GLP1 for each participant for both assessments (BL = baseline, FU = follow-up) divided by fasting status. **(G)** Longitudinal plot for participants with over-night fasting status (only assessed in the morning, *N* = 9). **(H)** Longitudinal plot for participants without over-night fasting status (assessed in the afternoon during BL or FU or both assessments, *N* = 8).
